# The prevalence and social-structural correlates of housing status among women living with HIV in Vancouver, Canada

**DOI:** 10.1186/s12889-022-14113-9

**Published:** 2022-09-22

**Authors:** Yinong Zhao, Kate Shannon, Jane A. Buxton, Lianping Ti, Theresa A. Genovy, Melissa Braschel, Kathleen Deering

**Affiliations:** 1 Faculty of Medicine, University of British Columbia, Vancouver, Canada; 2 Centre for Gender and Sexual Health Equity, Vancouver, BC Canada; 3 British Columbia Centre for Disease Control, Vancouver, Canada; 4 British Columbia Centre On Substance Use, Vancouver, Canada

**Keywords:** Women, HIV, Homelessness, Violence, Health

## Abstract

**Background:**

Women living with HIV (WLWH) experience numerous social and structural barriers to stable housing, with substantial implications for access to health care services. This study is the first to apply the Canadian Definition of Homelessness (CDOH), an inclusive national guideline, to investigate the prevalence and correlates of housing status among WLWH in Metro Vancouver, Canada.

**Methods:**

Our study utilized data from a longitudinal open cohort of cisgender and trans WLWH aged 14 years and older, in 2010–2019. Cross-sectional descriptive statistics of the prevalence of housing status and other social and structural variables were summarized for the baseline visits. Bivariate and multivariable logistic regression analyses were conducted using generalized linear mixed models (GLMM) for repeated measures to investigate the relationship between social and structural correlates and housing status among WLWH.

**Results:**

The study included 336 participants with 1930 observations over 9 years. Housing status derived from CDOH included four categories: unsheltered, unstable, supportive housing, and stable housing (reference). Evidence suggested high levels of precarious housing, with 24% of participants reporting being unsheltered, 47% reporting unstable housing, 11.9% reporting supportive housing, and 16.4% reporting stable housing in the last six months at baseline. According to the multivariable models, living in the Downtown Eastside (DTES) neighbourhood of Metro Vancouver, hospitalization, physical/sexual violence, and stimulant use were associated with being unsheltered, compared to stable housing; DTES residence, hospitalization, and physical/sexual violence were associated with unstable housing; DTES residence and stimulant use were associated with living in supportive housing.

**Conclusion:**

Complex social-structural inequities are associated with housing instability among WLWH. In addition to meeting basic needs for living, to facilitate access to housing among WLWH, housing options that are gender-responsive and gender-inclusive and include trauma- and violence-informed principles, low-barrier requirements, and strong connections with supportive harm reduction services are critical.

## Background

In North America, women comprise approximately one-third of all people who are unsheltered or living in unstable housing situations, and the proportion has been growing in the recent decade [1, 2]. Studies or programs that aim to count the number of people experiencing homelessness undercount women experiencing ‘hidden homelessness’, characterized by either living with family, friend, or abusive partner or staying in overcrowded and substandard housing to avoid unsheltered homelessness or co-ed homeless shelters [3]. Moreover, almost no studies disaggregate according to gender identity; most studies of women include cisgender (cis) women only, while trans women are often overlooked in discussions on homelessness [4]. The prevalence of episodic or chronic homelessness among women remains unclear. Improved methodology encompassing women’s housing experiences is urgently needed.

Housing has been identified as a basic human right and a critical social determinant of health [3, 5–7]. Homelessness and unstable housing among women have been associated with mortality, cardiovascular diseases, obesity, substance use, mental health conditions, injuries, and infectious diseases [6, 7]. The drivers of homelessness and unstable housing are complex. Women may choose to stay in precarious, violent housing situations or stay with relatives or friends rather than accessing emergency shelters due to multiple gender-based social-structural factors, including financial strain, childcare, and fear of gender-based violence [8, 9]. Gaetz et al.’s model (2013) suggested that inadequate systems (e.g., barriers to public funding, inadequate discharge planning) and structural inequities (e.g., income, discrimination, affordability and availability of housing) often fail to prevent individuals experiencing traumatic events, personal crisis, and health challenges from entering homelessness [9].

Markers of systemic and structural marginalization, including drug use, HIV, and poverty, have been shown to be linked to homelessness and unstable housing. Women experiencing homelessness had a ten-fold premature mortality relative to non-homeless counterparts, with HIV/AIDS and drug-related overdose being leading causes [7]. Low-rent Single Room Occupancy hotels (SROs) were found to have substandard living conditions, undermined tenancy rights, social violence, and gender-based violence towards women tenants, and they were the limited affordable housing for many PLWH (people living with HIV) and people who use drugs (PWUD) in Downtown Eastside (DTES), a Vancouver neighbourhood characterized by high levels of poverty and open drug scene [10]. Further, women with inadequate income experiencing perpetual evictions in urban settings lacking systemic, structural support to break the cycle of poverty and eviction [11]. Trans women face even more barriers to safe housing than cis women due to the exclusion, discrimination, and abuse based on their gender identities [12].

Women living with HIV (WLWH) are particularly marginalized amid housing and healthcare challenges. With limited research conducted with WLWH, housing has been identified as a critical determinant of HIV care continuum outcomes. A study with WLWH in San Francisco identified a dose–response relationship between more nights in unstable housing and homelessness and unsuppressed viral load [13]. Among PLWH who also use drugs in British Columbia (BC), homelessness was associated with unsuppressed viral load [14]. For PLWH taking antiretroviral therapy (ART), a dose–response relationship was found between longer homeless duration and lower likelihood of HIV viral suppression [14]. If the homeless individuals were hypothetically housed, modelling showed doubling in viral suppression among PLWH who also use drugs [15]. These findings have been explained by the association between lack of housing and delayed entry, poor access to HIV medical care, and poor quality and adherence to ART, subsequently resulting in unsuppressed viral load and mortality [5, 7]. However, studies on housing and eviction tend not to include a focus with women or stratify by gender, even though women often make up 30–40% of the study populations [14, 15]. Despite the findings of negative impacts of homelessness and unstable housing, there remains a knowledge gap in the prevalence of homelessness and other housing arrangements among WLWH. Limited evidence is available to guide the development of safe housing programs with and for WLWH.

Our study on the housing status among WLWH needed to address lack of appropriate definitions to include women’s experiences and the lack of consensus in the definitions of homelessness in current literature. We therefore referenced the Canadian Definition of Homelessness (CDOH), an inclusive national guideline by the Canadian Observatory on Homelessness [16], such that our study findings can include women’s experiences and be translatable to stakeholders nationally. Our main objectives are, amongst our study sample of WLWH in Metro Vancouver: (1) to estimate the prevalence of housing status categories aligned with the CDOH; (2) to identity the social-structural correlates of housing status among WLWH in Metro Vancouver.

## Methods

### Study population

Data collected in January 2010 to February 2019 were drawn from the Sexual Health and HIV/AIDS: Longitudinal Women’s Needs Assessment (SHAWNA). SHAWNA is an ongoing community-based study of WLWH (2014-present) which aims to understand the social and structural factors that shape access to health services among WLWH, including access to HIV treatment and care. Founded on extensive consultation with community, clinical, and policy experts, SHAWNA is committed to the GIPA/MIPA (Greater/Meaningful Involvement of People living with HIV) principle since conception. The SHAWNA community advisory board includes members of 15 + clinical, HIV, and community organizations.

Eligibility for SHAWNA includes cis and trans WLWH aged 14 + who primarily live and/or access HIV care in Metro Vancouver. The participants were recruited by Peer Research Associates, self-referrals, and referrals from HIV care providers, peer navigators, HIV/AIDS organizations, and clinical outreach. At baseline and every six months, the participants attended a questionnaire interview administered by community or peer interviewers and a clinical HIV and sexual health visit. The questionnaire collects socio-demographics and information regarding structural vulnerability and aspects of sexual and reproductive health access and HIV-related questions. All variables used in analysis were drawn from SHAWNA’s questionnaires. Survey items were chosen based on extensive community consultation with clinical and community organization collaborators, participants, peer researchers, Positive Women’s Advisory Board and the community advisory board, alongside the principal investigators and study staff. Twenty-seven percent of SHAWNA participants were also enrolled in An Evaluation of Sex Workers Health Access (AESHA) (2010-present), a cohort of sex workers in Metro Vancouver (≥ 14 years) [17].

Participants voluntarily undergo laboratory tests for HIV viral load, CD4, hepatitis C antibody, and sexually transmitted infections. Treatment and referral for active infection are made accordingly by a sexual health nurse. Each participant receives a $50 CAD compensation for each interview and lab visit for their time, travel, and expertise. SHAWNA holds ethical approval through Providence Health Care/University of British Columbia Research Ethics Board and BC Women’s Hospital. Data are securely collected and managed using REDCap electronic data capture tools hosted at the University of British Columbia [18].

### Primary outcome variable

**Fig. 1:**
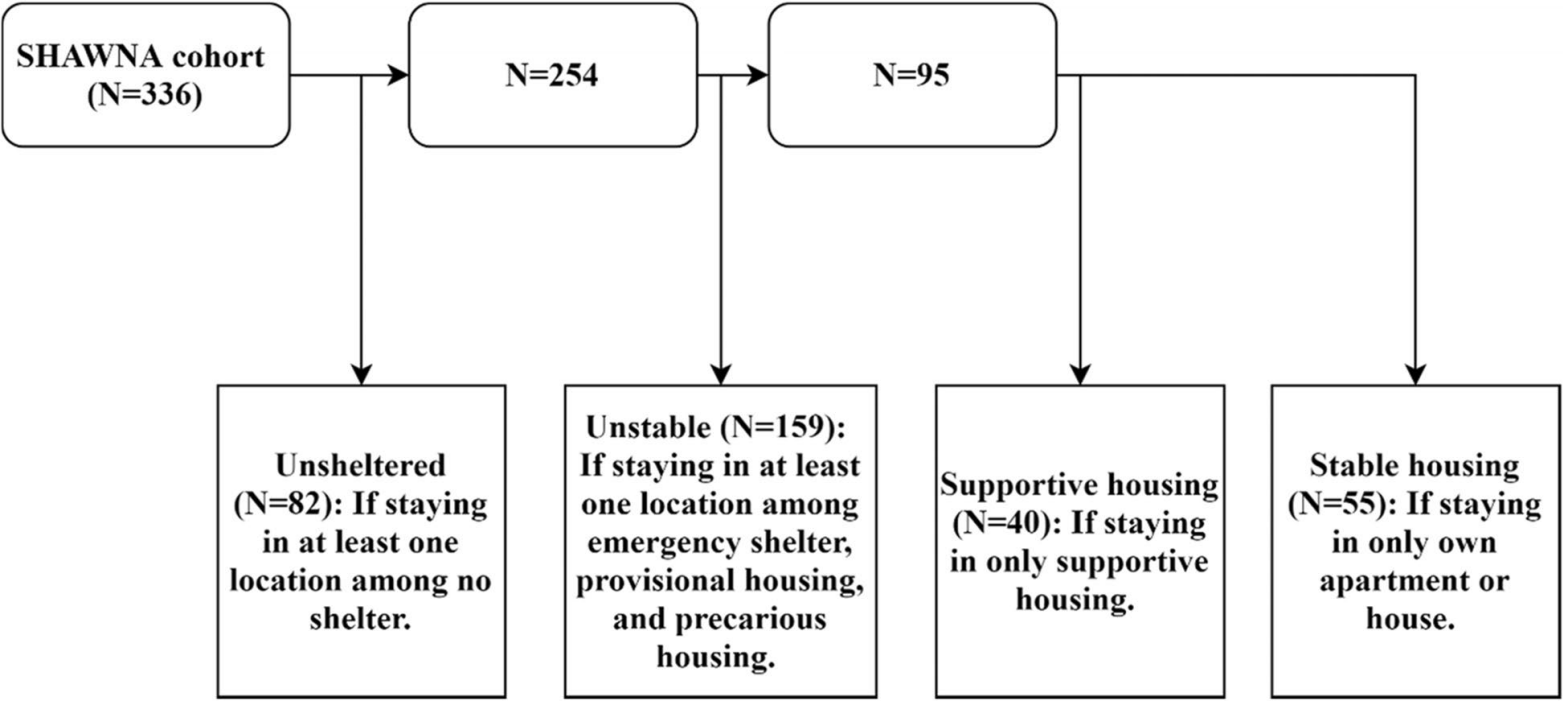
Step two of two: Characterizing housing status according to the participants’ all recent housing experience

Housing status as the primary outcome was defined according to the CDOH which considers homelessness as a dynamic state and recognizes various unsheltered and sheltered homeless situations [16]. Housing status was time-updated at each semi-annual study visit and was determined according to the types of places (one or more) where participants slept overnight. Over 50 types of locations were classified into six initial housing categories (Table 1). Due to multiple reported locations per participant, we further defined the housing status into four mutually exclusive categories (Fig. 1): (1) ‘unsheltered’; (2) ‘unstable’; (3) ‘supportive housing’; and (4) ‘stable housing’ (reference). The ‘unsheltered’ and ‘unstable’ categories intentionally capture individuals who have stayed in multiple accommodations to reflect the complexity and instability of their housing situations. For example, a combination of living in a car, staying with friends, and supportive housing would be defined as ‘unsheltered’, using the least stable categorization. Similarly, staying with friends and supportive housing would be defined as ‘unstable’.

**Table 1:** Step one of two: characterizing housing status. Descriptions and examples of the initial six housing categories

Initial Housing Categories	Descriptions and Examples
No shelter	Living on the street, in vehicles, in abandoned buildings, and anywhere that is not designed or fit for habitation
Emergency shelter	Staying at an emergency shelter due to extreme weather, violence, natural disaster, and so on
Provisional housing	Staying with family and friends, staying at interim housing for the homeless, being in institutional care and lacking permanent housing arrangements. The key feature is lacking the security of tenure of housing
Precarious housing	Staying at Single-Room Occupancy (SRO) hotels
Supportive housing	Staying at any supportive housing recognized by the provincial government, HIV-specific supportive housing, and non-profit housing for those with special needs
Own apartment or house	Staying at one’s own apartment or house alone or with family, intimate partner, and roommates

### Sociodemographic and explanatory variables

Time-fixed social-structural variables included: race (Indigenous [First Nations, Metis, Inuit], other racialized women [African, Caribbean, Black, Latin American, Asian, other], vs. only reporting white); highest level of education completed (high school level and above [high school graduate, any college/university, trade, GED] vs. below high school level); sexual orientation (sexual minority at any study visit [inclusive of lesbian, gay, bisexual, asexual, Two-Spirit, queer, other] vs. only heterosexual at all study visits); and gender identity (gender minority at any study visit [inclusive of trans [transgender, transsexual, other transfeminine identity], non-binary [non-binary, genderqueer], Two-Spirit] vs only cisgender at all visits). The term Indigenous is used throughout while recognizing the great diversity across and within languages, cultures, nations and lands. While descriptive data were disaggregated, given small sample size of Black participants, compared to the BC population, Black/other racialized women of colour were combined. Indigenous women were asked if they identified as Two-Spirit. Two-Spirit is an identity among people Indigenous to Turtle Island who identify as having both a masculine and a feminine spirit, and may be used to describe any or all of sexual, gender and/or spiritual identity; however, this depends on the individual and context [19]. Participants had the option to provide more than one response to questions on sexual orientation and gender identity. Based on evidence that minority stress processes affect gender minority people relative to cis people [20], and sexual minority people relative to heterosexual people [21], and given limited sample size, for the purposes of analyses, we combined participants with responses to sexual minority identities into one variable, and participants with gender minority identities into one variable.

All other variables were time-updated at each semi-annual study visit. Time-updated social-structural variables that were measured in a ‘current’ timeframe included: age (measured continuously, in years); location of residence (City of Vancouver vs. not City of Vancouver; DTES vs. not DTES). Time-updated social-structural variables capturing events in the last six months included: employment (formal, legal employment, sex work vs. none or nonlegal employment); average monthly income (including government allowances, in $CAD); food insecurity (measured by a version of the Radimer/Cornell Hunger Scale; ‘often true’ or ‘sometimes true’ to at least one item vs. ‘never true’ or ‘not applicable’ to all items) [22]. Institutionalization variables included: lifetime incarceration (time-updated); hospitalization in the last six months. All behavioural variables captured events in the last six months and included: any stimulant drug use; any opioid drug use; drug overdose from any substance. Interpersonal variables included: feeling in danger where currently sleeping; experience of physical/sexual violence in the last six months (by any perpetrator); ever being outed as HIV positive (time-updated); ever being abused due to HIV status (time-updated).

### Statistical analysis

Cross-sectional descriptive sample characteristics were calculated to examine sociodemographic variables stratified by housing categories at baseline. Categorical variables were summarized as frequencies and proportions, and continuous variables as medians and first to third quartile (Q1-Q3). P-values were calculated using Pearson’s chi-square test for categorical variables (or Fisher’s exact test for small cell counts) and analysis of variance (ANOVA) for continuous variables. Using longitudinal data, bivariate and multivariable generalized linear mixed models (GLMM) were used to examine associations with the multinomial outcome using a generalized logit link; random intercepts were incorporated to account for to account for between- and within-subject variability of repeated measures (including time-varying variables, such as housing status) among participants. Variables that had strong bivariate associations (*p* < 0.10) with any housing category were considered for inclusion in the multivariable explanatory model. Backward stepwise model selection was used to determine the model with the best fit, as indicated by the lowest Akaike Information Criterion. A complete case approach was used such that rows with missing data were excluded from analysis. This resulted in 1.2% (*n* = 4) participants excluded from the multivariable. Odds ratios (OR), adjusted odds ratios (AOR) and 95% confidence intervals (CI) were presented; all *p*-values were two-sided. All analyses were performed in SAS version 9.4 (SAS Institute Inc., Cary, North Carolina, USA).

## Results

The study sample included 1930 observations on 336 participants over 9 years, who contributed a median of 5 study visits (Q1-Q3: 3–7). At baseline, 7.1% (24) of participants reported trans identity, and 92.9% (312) were cis. A total of 34.8% (117) of participants reported sexual and/or gender minority identity, 32.7% (110) of participants reported sexual minority identity with 9.8% (33) reporting gender minority identity. Recognizing fluidity in gender identity over time, 1.5% (5) reported non-binary gender identity at some point in the study. Indigenous women were overrepresented in this sample at 56.9% (191) relative to the population of British Columbia (5.9% in 2016) [23]; 12.6% (24) of Indigenous participants were Two-Spirit. The sample also included 5.4% (18) Black women, 3.6% (12) otherwise racialized women, and 34.2% (115) white woman. In the last six months, 71.7% (241) of participants reported living in unsheltered (24.4%, *n* = 82) and unstable (47.3%, *n* = 159) housing situations; 28.3% (95) lived in either supportive housing (11.9%, *n* = 40) or their own apartment or house (16.4%, *n* = 55). Please see Table 2 for additional characteristics of the study sample.

**Table 2:** Baseline demographics and characteristics of 336 WLWH from SHAWNA cohort stratified by the housing status

			Housing status				
	Total *N* = 336 (100)	Missing data (%)	Unsheltered *N* = 82 (24.4)	Unstable *N* = 159 (47.3)	Supportive housing *N* = 40 (11.9)	Stable housing *N* = 55 (16.4)	*P*-value
Age (median, Q1-Q3)	43 (36–50)	0 (0)	40 (34–46)	44 (37–52)	48 (43–53)	44 (36–50)	< 0.001
Sexual minority	110 (32.7)	1 (0.3)	26 (31.7)	56 (35.2)	12 (30.0)	16 (29.1)	0.836
Gender minority	33 (9.8)	2 (0.6)	–	–	–	–	0.008
Race White Indigenous Otherwise racialized	115 (34.2) 191 (56.9) 30 (8.9)	0 (0)	– – –	– – –	– – –	– – –	0.384
Currently live in City of Vancouver	246 (73.2)	1 (0.3)	–	–	–	–	< 0.001
Currently live in DTES	103 (30.7)	1 (0.3)	43 (52.4)	36 (22.6)	18 (45.0)	6 (10.9)	< 0.001
Education, high school level and above	161 (47.9)	0 (0)	29 (35.4)	87 (54.7)	16 (40.0)	29 (52.7)	0.022
Employment^a^ None Sex work Formal, legal	165 (49.1) 115 (34.2) 44 (13.1)	12 (3.6)	– – –	– – –	– – –	– – –	0.001
Monthly income in $CAD^a^ (median, Q1-Q3)	1,600 (1,110–2,660)	5 (1.5)	1,490 (1,000–3,150)	1,700 (1,140–2,820)	1,380 (1,110–1,930)	1,690 (1,180–2,400)	0.085
Food insecurity^a^	260 (77.4)	2 (0.6)	71 (86.6)	123 (77.4)	27 (67.5)	39 (70.9)	0.077
Incarceration^b^	246 (73.2)	1 (0.3)	66 (80.5)	113 (71.1)	32 (80.0)	35 (63.6)	0.081
Hospitalization^a^	79 (23.5)	1 (0.3)	–	–	–	–	< 0.001
Stimulant use^a^	221 (65.8)	1 (0.3)	72 (87.8)	93 (58.5)	31 (77.5)	25 (45.5)	< 0.001
Opioid use^a^	143 (42.6)	1 (0.3)	49 (59.8)	60 (37.7)	17 (42.5)	17 (30.9)	0.003
Overdose^a^	19 (5.7)	3 (0.9)	–	–	–	–	0.257
Feel in danger where currently sleeping	89 (26.5)	0 (0)	26 (31.7)	44 (27.7)	9 (22.5)	10 (18.2)	0.316
Physical/sexual violence^a^	62 (18.5)	15 (4.5)	–	–	–	–	< 0.001
Outed as HIV + ^b^	154 (45.8)	11 (3.3)	38 (46.3)	69 (43.4)	17 (42.5)	30 (54.6)	0.686
Abused due to HIV status^b^	108 (32.1)	21 (6.3)	32 (39.0)	50 (31.5)	11 (27.5)	15 (27.3)	0.383

All data refer to n (%) of participants unless otherwise specified

Q1-Q3: first to third quartile

Low cell counts (< 5) are suppressed to maintain participant privacy

^a^ Last six months prior to the interview

^b^ Lifetime

In bivariate analysis, the following variables were significantly associated with housing status at a *p* < 0.10-level: age, sexual minority, gender minority, living in DTES, food insecurity, employment, incarceration, hospitalization, stimulant use, opioid use, and physical/sexual violence. Multivariable analysis identified the following variables associated with being unsheltered or unstable housing versus stable housing (Table 3): for being unsheltered, age (AOR = 0.96 per year older, 95%CI (0.93–0.99)), DTES residence (AOR = 5.22, 95%CI (3.06–8.90)), sex work (AOR = 2.58, 95%CI (1.11–6.00)), hospitalization (AOR = 4.93, 95%CI (2.66–9.12)), stimulant use (AOR = 2.69, 95%CI (1.56–4.61)), and physical/sexual violence (AOR = 4.71, 95%CI (2.56–8.68)); for unstable housing, living in DTES (AOR = 2.20, 95%CI (1.42–3.43)), hospitalization (AOR = 7.86, 95%CI (4.65–13.30)), and physical/sexual violence (AOR = 3.00, 95%CI (1.76–5.13)); for supportive housing, age (AOR = 1.04 per year older, 95%CI (1.01–1.07)), living in DTES (AOR = 3.30, 95%CI (1.94–5.60)), incarceration (AOR = 2.21, 95%CI (1.13–4.34)), and stimulant use (AOR = 2.32, 95%CI (1.42–3.77)). The following variables were included in the full multivariable model, but not retained as significantly associated with housing status after the model fitting process: sexual minority, gender minority, food insecurity, employment, and opioid use.

**Table 3:** Unadjusted and adjusted odds ratios and 95% confidence intervals (CI) from bivariate and multivariable GLMM for significant correlates of housing status

	Unadjusted Odds Ratio (95% CI)			Adjusted Odds Ratio (95% CI)		
	Unsheltered	Unstable	Supportive Housing	Unsheltered	Unstable	Supportive Housing
Age (per year older)	0.91 (0.88–0.95)***	1.00 (0.98–1.02)	1.02 (0.99–1.06)	0.96 (0.93–0.99)**	1.01 (0.99–1.04)	1.04 (1.01–1.07)*
Sexual Minority^a^	2.16 (1.21–3.87)**	1.41 (0.95–2.10)	1.26 (0.69–2.28)			
Gender minority^a^	2.32 (0.99–5.42)	0.69 (0.38–1.25)	0.71 (0.28–1.78)			
Race^b^ Indigenous Otherwise racialized	1.20 (0.65–2.23) 0.42 (0.13–1.39)	1.01 (0.67–1.53) 0.81 (0.39–1.68)	1.16 (0.63–2.14) 0.48 (0.15–1.53)			
Currently live in DTES	7.72 (4.71–12.66)***	2.58 (1.72–3.89) ***	3.61 (2.20–5.93) ***	5.22 (3.06–8.90)***	2.20 (1.42–3.43)***	3.30 (1.94–5.60)***
Food insecurity^a,c^	1.58 (1.02–2.45)*	1.14 (0.82–1.57)	1.04 (0.68–1.57)			
Employment^c,d^ None Sex work	2.02 (0.98–4.20) 7.09 (3.22–15.62)***	0.71 (0.45–1.10) 1.53 (0.91–2.56)	1.23 (0.67–2.28) 1.52 (0.75–3.06)	1.16 (0.55–2.46) 2.58 (1.11–6.00)*	0.57 (0.36–0.90)* 1.16 (0.65–2.07)	1.21 (0.64–2.28) 1.23 (0.57–2.65)
Incarceration, lifetime	3.00 (1.54–5.83)**	1.91 (1.25–2.91)**	3.15 (1.61–6.15)***	1.28 (0.63–2.62)	1.31 (0.83–2.05)	2.21 (1.13–4.34)*
Hospitalization^c^	4.32 (2.47–7.57)***	7.20 (4.43–11.71)***	1.00 (0.54–1.84)	4.93 (2.66–9.12)***	7.86 (4.65–13.30)***	1.07 (0.55–2.06)
Stimulant use^c^	5.77 (3.63–9.18)***	1.78 (1.29–2.47)***	3.01 (1.96–4.62)***	2.69 (1.56–4.61)***	1.06 (0.72–1.55)	2.32 (1.42–3.77)***
Opioid use^a,c^	3.63 (2.35–5.62)***	1.51 (1.08–2.11)*	1.74 (1.15–2.64)**			
Overdose^c^	3.07 (1.38–6.81)**	1.81 (0.89–3.68)	1.37 (0.60–3.14)			
Physical/sexual violence^c^	6.67 (3.77–11.83)***	3.12 (1.88–5.18)***	1.74 (0.94–3.22)	4.71 (2.56–8.68)***	3.00 (1.76–5.13)***	1.62 (0.86–3.05)

The stable housing category is the reference for all odds ratios

^*^p < 0.05

^**^p < 0.01

^***^p < 0.001

^a^ Variable was included in the full multivariable explanatory model but not retained in the best fitting model

^b^ White is the reference

^c^ Time-updated variable capturing events in the last six months at each semi-annual study visit

^d^ Formal, legal employment is the reference

## Discussion

Using the CDOH, our study identified that most WLWH in our Metro Vancouver cohort had experienced unsheltered and unstable living situations in the last six months at baseline. Key social-structural factors, including living in the Downtown Eastside (DTES) neighbourhood of Metro Vancouver, hospitalization, physical and/or sexual violence in the last six months, were associated with unsheltered and unstable housing (vs. stable housing), with important implications for housing to address and prevent homelessness among WLWH.

Among WLWH in our study, 24.4% reported unsheltered housing situations and 47.3% reported unstable housing in the last six months at their baseline interview, with an overall 71.7% reported living in unsheltered and unstable housing (Table 2). We found limited studies with which to compare the prevalence of precarious housing among WLWH in other settings. One study of PLWH revealed 8.1% prevalence of homelessness and SRO residence among WLWH and 19.6% among trans PLWH [24] Further, the combined prevalence in our study sample is also higher than the prevalence of housing insecurity (measured by asking participants if they have difficulty affording housing and related costs) reported in a Canadian study with WLWH, at 51.5% [25]. The discrepancy may be due to the differences in how housing status was measured. In comparison to other definitions of homelessness, measuring housing status according to the CDOH provides a more comprehensive assessment of the housing status and offers categorizations that encompass the complexity of participants’ housing experiences.

DTES residence was associated with over five times the odds of being unsheltered and over twice the odds of unstable housing. These associations seem reasonable in the context of overlapping experiences with marginalization and poverty among WLWH. Vancouver’s DTES has been experiencing a systemic and structural housing crisis [26, 27]. The inexpensive and often precarious rental options and an accepting culture attracted many marginalized and low-income populations [26]. Widely available criminalized drugs have also contributed to exacerbating poverty and displacing people from housing intolerant of drug use [26]. With a growing number of higher-income urban developments, recent decades saw a systemic decrease in cheap rental units, further limiting the housing options [26, 27]. Low-rent SROs lack adequate living space, maintenance, and tenancy right protection [10]. While the resilient and vibrant community has been supported by grassroot organizations, interventions from provincial and municipal government are required along meaningful consultation with community members to increase the availability of affordable and humane housing options.

Recent stimulant use was associated with almost three times the odds of being unsheltered. Substance use as a coping mechanism in response to trauma [28]. Substance use is a known contributor to the loss of housing due to financial instability, social stigma and limited options for low-barrier approaches to drug use [9, 28, 29]. Meanwhile, experiencing homelessness can lead to or increase substance use for coping stress, resulting in a cycle of homelessness and substance use [28]. Further, historical and current colonial violence and trauma, including the devastatingly negative effects of residential schools, have impacted generations of Indigenous people, resulting in disproportionate prevalence of substance use and addiction [29]. The stigmatization of substance use and addiction fuels discrimination against potential tenants perceived to use drugs, which limits access to rental housing [30]. For PWUD to gain access to stable long-term housing, housing programs need to follow harm-reduction principles and provide or link to adequate, culturally safe and gender-responsive, gender-inclusive treatment programs with trauma- and violence-informed (TVI) principles.

Our study identified an association between hospitalization and homelessness. Current literature has conceptualized homelessness and unstable or precarious housing as a reason to explain increased utilization of the emergency department among PLWH in BC, whereas stable housing can encourage connection with primary care to avoid misuse [31]. Meanwhile, hospitalization could also lead to and explain homelessness or unstable housing [32]. Systemically, inadequate discharge planning could introduce someone with limited resources to homelessness [9]. On a structural level, hospitalization could limit income generation and lead to unemployment, poverty, and homelessness [9]. On the individual level, severe and debilitating illnesses could prevent securing employment and housing. Individuals with mental health conditions might experience disruption of social connections from family and others that support stable housing [32]. Homelessness and/or unstable housing has been associated with higher levels of hospitalization in other settings, with women experiencing homelessness and/or unstable housing having 3.5 times the rate of hospitalizations and 11.9 times the rate of outpatient mental health and substance use service usage events relative to the general population of women [33, 34]. WLWH in the United States had 51% higher rates of hospitalization than MLWH, while PLWH had higher likelihoods of hospitalization than the general population [35]. For trans WLWH, the lack of gender-responsive and gender-inclusive care further limited healthcare access [36]. Limited healthcare access while being homeless can exacerbate HIV and other illnesses, resulting in a cycle of worsening health and homelessness [5, 32]. Housing developments and public-funded programs with WLWH should facilitate healthcare access and provide adequate financial and personal support to prevent homelessness.

Our findings were consistent with existing literature that gender-based violence is a major cause of homelessness among women [9], and these effects may be exacerbated for racialized WLWH and WLWH with minoritized and marginalized sexual and/or gender identities. Women and children are most likely to become homeless upon leaving violent relationships or households [3, 9]. In Canada, the destructive effects of structural racism, residential schools and other colonial violence has resulted in Indigenous women experiencing high levels of interpersonal violence and homelessness [30]. In New Zealand, colonial violence has similarly driven the over-representation of Indigenous Maori women among women experiencing unstable housing, with nearly 80% of Indigenous Maori women experiencing unstable housing nationwide, 5.5 times of the general population [33, 34]. In an Ontario study of gender-diverse people, 73% ever experienced violence, and 67% reported having to move due to their gender identity and expression (35). Even at women-specific shelters, trans and two-spirit individuals are subject to structural violence, including discrimination, social exclusion, and gender policing [12]. HIV-related stigma exposes WLWH to verbal, physical, and sexual violence by any perpetrator, ranging from intimate partners to strangers [37]. Violence and trauma lead to psychological stress, damaged self-esteem, suicidality, and substance use [37], further marginalizing WLWH and contributing to housing instability. Our findings highlight the need to create safe, inclusive, and TVI housing solutions for all WLWH.

Inequities in employment and income among WLWH undermine housing opportunities. Unemployment, nonlegal employment, and sex work were prevalent among women in our study sample (Table 2). On a structural level, limited opportunities for secure income sources present barriers to accessing housing in over-inflated housing markets with high rents and low vacancies, resulting in loss of housing [9, 11, 29, 33]. One study suggested that the average income among women experiencing homelessness and unstable housing was less than one-fifth of the population average [33]. Another study observed that women experiencing heightened evictions had to spend 80–90% of income from minimum wage jobs on rent payments [11]. Lower education level not only limits the opportunities for employment and income, but also is linked to poor literacy and numeracy skills, which negatively affects securing housing [29]. Further, a racialized unemployment rate and pay gap affect income and housing status in some settings [38]. Intersectionality of race and socioeconomic status have been found to be associated with experiences of discrimination in securing housing among racialized and Indigenous Peoples [3]. Sufficient and timely income support is necessary to maintaining housing and bridge the long-standing income inequality for WLWH experiencing or at risk of homelessness. Meanwhile, additional programs should be designed with and for WLWH to introduce educational and recreational activities to build communities and encourage future engagement in the workforce.

Applying TVI approaches, gender-responsive and gender-inclusive supportive housing programs involving WLWH in design and implementation should be tailored for and with WLWH with critical housing and healthcare needs. To provide stable housing, reduce structural inequities, and support healthcare access, such programs often featured case management, peer support, cultural safety, harm reduction practices, and supports for maintaining family bonds [30, 39, 40]. Programs should be adaptable in fostering connection with cultural and traditional practices to improve WLWH’s mental and spiritual wellbeing [30]. WLWH in supportive housing programs may have increased chance of achieving viral suppression than those in conventional care [40]. The Housing First model may align with the housing needs of some WLWH by providing stable housing, harm reduction, supportive staff, privacy, and physical security [41, 42]. These features have been found to contribute positively towards the overall improvement in residents’ physical and mental health [41, 42]. Globally and in Canada, Housing First programs have provided stable housing to urban populations who experience homelessness and have mental health conditions [33, 34, 41]. Meanwhile, gaps in the Housing First model have been identified and need to be addressed to meet the needs of WLWH [43]. For example, limited recruitment of women and single-mother families has been observed in the programs [41–43]. Women’s lack of representation in Housing First might have resulted from a failure to consider and incorporate drivers of women’s loss of housing, including gender-based violence, the needs of larger family-size housing, and the lack of women-specific/women-only housing [43]. Additionally, Housing First programs rely on existing affordable housing, rather than creating new affordable options [42]. Therefore, housing programs must also be supported by concurrent policy to increase the number of affordable housing units and provide means (e.g., adequate income, employment opportunities) in order to address the root causes of homelessness and unstable housing among WLWH [33, 42].

Our study has several limitations and strengths. Self-reported data might introduce recall and reporting biases, but the community-based nature of SHAWA is designed to mitigate this. This study cannot infer causality; findings are likely not generalizable to all WLWH in Canada. Though longitudinal data collection increases statistical power via repeated measures, the sample size may have precluded us from detecting some associations, due to the exploratory nature of the study. There were significant differences in housing status according to gender identity in bivariate analysis, but this association was not retained in multivariable analysis. Low sample sizes among women with gender minority identities in our study may have affected our ability to detect the associations in multivariable analyses. A major strength was being the first study to reference the CDOH in defining the housing status among WLWH and categorizing 50 + types of accommodation into a four-category variable capturing complexity and diversity. By using the CDOH, our findings will be easily translatable to knowledge users in housing research and policy nationwide.

To conclude, our study highlighted the prevalent housing instability among WLWH in Metro Vancouver, Canada. Homelessness among WLWH is a complex product of systemic and structural inequities. Our results echo the need for interventions for WLWH and other marginalized populations to protect their basic right to housing. The experience, concerns, and needs of WLWH must be consulted to resolve the housing crisis. Structural inequity and marginalization experienced by a diverse WLWH population need to be addressed to achieve stable housing, as well as financial security, physical wellbeing, freedom from violence and discrimination to prevent future homelessness. Results from our study suggest that housing options for WLWH that are gender-responsive, gender-inclusive, low-barrier, and incorporate TVI, harm reduction, and cultural safety practices are critical to support women in accessing housing. An important future direction would be to examine the role of housing in the healthcare access and HIV care continuum among WLWH. Further research is needed to understand the housing needs of WLWH with marginalized and minoritized gender identities, given high levels of discrimination and violence [12, 36] that may affect their access to safe and stable housing and few gender-responsive and gender-inclusive programs developed specifically to meet their needs. With further evidence, a stronger case will be made to protect the housing rights of WLWH.

## Data Availability

The datasets generated and/or analysed during the current study are not publicly available due to our ethical and legal requirements related to protecting participant privacy and current ethical institutional approvals but are available from the corresponding author on reasonable request pending ethical approval.
